# Prediction of surgical margin status and location after radical prostatectomy using positive biopsy sites on 12-core standard prostate biopsy

**DOI:** 10.1038/s41598-022-08022-5

**Published:** 2022-03-08

**Authors:** Hyeon Jeong, Min Soo Choo, Min Chul Cho, Hwancheol Son, Sangjun Yoo

**Affiliations:** Department of Urology, Seoul National University Boramae Medical Center, Sindaebang 2(i)-dong, Dongjak-gu, Seoul, 07061 Korea

**Keywords:** Cancer, Urogenital diseases

## Abstract

We evaluated the surgical margin status after radical prostatectomy according to sites positive for prostate cancer on standard 12-core transrectal ultrasound-guided prostate biopsy. Among patients who underwent radical prostatectomy at Boramae Medical Center, 520 patients with preoperative prostate-specific antigen (PSA) level < 20 ng/mL and locally confined prostate cancer on preoperative magnetic resonance imaging, treated with nerve-sparing radical prostatectomy, were included in the analysis. The surgical margin was positive for cancer in 166 (31.9% of the total) patients. The preoperative PSA level (9.3 vs. 8.0, ng/mL *p* = 0.001) and number of positive cores on 12-core prostate biopsy (4.1 vs. 3.4, *p* = 0.003) were significantly higher in patients with positive surgical margins. Moreover, the biopsy Gleason grade was higher in patients with positive surgical margins (*p* = 0.001). However, the pathologic Gleason grade and tumor volume were equivalent between the 2 groups. On multivariate analysis, the detection of prostate cancer on anterior lateral biopsy was associated with an increased rate of positive surgical margins (hazard ratio [HR]: 1.781, *p* = 0.008) after adjusting for other variables. Anterior lateral (HR: 1.919, *p* = 0.020), basal lateral (HR: 9.176, *p* < 0.001), basal medial (HR: 3.302, *p* = 0.031), and mid lateral (HR: 2.501, *p* = 0.044) biopsies were associated with positive apical, posterior, basal, and lateral surgical margins, respectively, after adjusting for other variables. The sites of prostate cancer on standard 12-core prostate biopsy could be useful for predicting surgical margin positivity after radical prostatectomy. In other words, clinicians should consider the sites of prostate cancer on prostate biopsy to reduce margin positivity after radical prostatectomy.

## Introduction

Prostate cancer is the second most common cancer in men worldwide^1^, and approximately 70–80% of prostate cancer cases are localized disease at the time of diagnosis^2^. Radical prostatectomy has been considered the gold standard treatment method for localized prostate cancer^3^. However, recently, the treatment methods for localized prostate cancer have become more diversified based on tumor and patient characteristics, and active surveillance has become a preferred treatment option for very low-risk prostate cancer^3^. In other words, unlike before, radical prostatectomy is now preferably offered to patients with more aggressive prostate cancer, which has a higher probability of postoperative recurrence.

The recurrence of prostate cancer after radical prostatectomy is influenced by several factors^4^. Among them, surgical margin status is one of the most important for predicting oncologic outcomes^5^. To minimize surgical margin positivity, the anatomical characteristics of the prostate have been investigated in detail and several surgical techniques have been introduced^6–8^. However, despite these improvements, the postoperative surgical margin is still reported to be positive for prostate cancer in approximately 20–30% of cases^9^.

The surgical margin status could be affected by several factors, including surgeon experience, surgical techniques, tumor characteristics, and anatomical characteristics^10,11^. In addition to these factors, the time period from prostate biopsy to radical prostatectomy also affects the surgical margin status because of biopsy-induced adhesive changes^12^. Moreover, the location of prostate cancer also affects the surgical margin status^13^ and the location of the positive surgical margin (PSM) in the final pathology. In other words, a positive prostate biopsy result at a certain location could predict a PSM status owing to the proximity to the incision site.

Surgical margin status is considered to be especially important in locally confined prostate cancer because this type of cancer has a high probability of complete removal if a negative pathologic surgical margin is achieved. In other words, if the probability for surgical margin positivity and its location could be appropriately addressed before surgery, the oncological outcomes in these patients could be greatly improved. Therefore, in this study, we aimed to evaluate the impact of positive prostate biopsy locations on the surgical margin status in the final pathologic outcomes after radical prostatectomy for clinically localized prostate cancer. In addition, we also sought to evaluate the relationship between positive prostate biopsy locations and the sites of PSM on the final pathologic examination.

## Results

After radical prostatectomy, the surgical margin was positive for prostate cancer in 166 (31.9%) patients with preoperative PSA level < 20 ng/mL and clinically localized prostate cancer (Table 1). The tumor volume (6.8 vs. 7.4 mL, *p* = 0.599) and percent tumor volume (15.4% vs. 18.4%, *p* = 0.130) on the final pathologic examination were equivalent regardless of the surgical margin status (Table 2). The pathologic Gleason grade was also equivalent regardless of the surgical margin status. Extracapsular extension was pathologically identified in 171 (33.3%) patients, and seminal vesicle invasion was identified in 75 (14.6%) patients. Extracapsular extension was significantly more frequent (40.1% vs. 30.2%, *p* = 0.027) and seminal vesicle invasion was marginally more frequent (18.5% vs. 12.8%, *p* = 0.090) in the PSM group.

**Table 1:** Baseline characteristics according to surgical margin status.

	NSM	PSM	*p*
Number of patients, n (%)	354 (68.1)	166 (31.9)	
Age, years, mean ± SD	67.3 ± 5.9	67.6 ± 6.6	0.574
BMI, kg/m^2^, mean ± SD	23.9 ± 2.7	24.3 ± 2.5	0.135
Hypertension, n (%)	178 (50.3)	92 (55.4)	0.274
Diabetes, n (%)	56 (15.8)	32 (19.3)	0.327
PSA, ng/mL, mean ± SD	8.0 ± 3.9	9.3 ± 4.3	0.001
Prostate volume, mL, mean ± SD	36.4 ± 16.6	33.5 ± 13.9	0.061
Days from biopsy to surgery, mean ± SD	45.4 ± 58.0	37.6 ± 24.5	0.112
Number of positive cores, n, mean ± SD	3.4 ± 2.4	4.1 ± 2.7	0.003
**MR findings**			
Visible focal lesions, n (%)	110 (31.1)	82 (49.4)	< 0.001
+Size of focal lesion	11.6 ± 5.8	13.3 ± 6.0	0.211
*Location of focal lesions			< 0.001
Peripheral zone	93 (85.3)	72 (88.9)	
Transition zone	15 (13.8)	5 (6.2)	
Both	1 (0.9)	4 (4.9)	
**Biopsy Gleason grade, n (%)**			0.001
1	170 (74.9)	57 (25.1)	
2	71 (68.9)	32 (31.1)	
3	48 (60.8)	31 (39.2)	
4	47 (60.3)	31 (39.7)	
5	7 (35.0)	13 (65.0)	
**Surgical methods, n (%)**			0.295
Open	180 (70.6)	75 (29.4)	
Minimal invasive	169 (66.3)	86 (33.7)	
**Surgeon, n (%)**			0.809
A	254 (67.7)	121 (32.3)	
B	63 (70.8)	26 (29.2)	
C	37 (66.1)	19 (33.9)	

*NSM* negative surgical margin, *PSM* positive surgical margin.

*Location of focal lesion on MR imaging was reported in 190 cases among 192 cases.

+Size of focal lesion on MR imaging was reported in 86 cases among 192 cases.

**Table 2:** Pathologic characteristics according to surgical margin status.

	NSM	PSM	*p*
Tumor volume, mL, mean ± SD	6.8 ± 11.8	7.4 ± 10.6	0.599
Pathologic tumor volume, %, mean	15.4 ± 20.8	18.4 ± 20.2	0.130
**Pathologic Gleason grade, n (%)**			0.168
1	106 (74.1)	37 (23.0)	
2	111 (68.5)	51 (31.5)	
3	81 (66.4)	41 (33.6)	
4	36 (67.9)	17 (32.1)	
5	16 (51.6)	15 (48.4)	
Pathologic extracapsular extension, n (%)	106 (30.2)	65 (40.1)	0.027
Pathologic seminal vesicle invasion, n (%)	45 (12.8)	30 (18.5)	0.090

NSM: negative surgical margin, PSM: positive surgical margin.

The percent tumor volume was significantly higher in patients with a positive AM biopsy, although it was equivalent regardless of tumor positivity in other sites of prostate biopsy (Table 3). Positive apical surgical margins were commonly observed in men with positive AM (17.4% vs. 10.5%) and AL (17.6% vs. 10.6%) biopsies. Meanwhile, positive basal surgical margins were commonly observed in men with a positive BM biopsy (6.3% vs. 2.7%). Positive anterior surgical margins were commonly observed in men with positive ML (11.2% vs. 5.9%), BM (12.0 vs. 6.6%), and BL (11.4 vs. 6.2%) biopsies. Positive posterior surgical margins were commonly observed in men with positive AL (7.6% vs. 3.4%), ML (8.5% vs. 2.6%), BM (8.3% vs. 3.9%), and BL (10.1% vs. 1.7%) biopsies. Positive lateral surgical margins were commonly observed in men with a positive BM biopsy (8.9% vs. 4.2%). The rate of positive posterolateral surgical margins was equivalent regardless of the site of prostate cancer detection on prostate biopsy.

**Table 3:** Site-specific surgical margin status and tumor volume according to the site of positive biopsy for prostate cancer.

Site	Surgical Margin	% tumor volume mean ± SD	Site of surgical margin, n (%)					
			Apex	Base	Ant	Post	Lateral	Posterolateral
AM	NSM	14.1 ± 19.1	27/257 (10.5)	12/257 (4.7)	17/257 (6.6)	11/257 (4.3)	14/257 (5.4)	3/257 (1.2)
	PSM	18.3 ± 21.7	47/270 (17.4)	9/270 (3.3)	28/270 (10.4)	18/270 (6.7)	17/270 (6.3)	2/270 (0.7)
	*p*	0.020	0.023	0.433	0.123	0.230	0.679	0.614
AL	NSM	15.9 ± 22.0	28/265 (10.6)	8/265 (3.0)	18/265 (6.8)	9/265 (3.4)	11/265 (4.2)	2/265 (0.8)
	PSM	16.5 ± 19.0	46/262 (17.6)	13/262 (5.0)	27/262 (10.3)	20/262 (7.6)	20/262 (7.6)	3/262 (1.1)
	*p*	0.742	0.021	0.254	0.149	0.033	0.089	0.644
MM	NSM	16.5 ± 21.9	35/284 (12.3)	10/284 (3.5)	19/284 (6.7)	11/284 (3.9)	16/284 (5.6)	1/284 (0.4)
	PSM	15.9 ± 18.9	39/243 (16.0)	11/243 (4.5)	26/243 (10.7)	18/243 (7.4)	15/243 (6.2)	4/243 (1.6)
	*p*	0.733	0.220	0.556	0.101	0.076	0.793	0.127
ML	NSM	15.6 ± 21.0	37/269 (13.8)	9/269 (3.3)	16/269 (5.9)	7/269 (2.6)	11/269 (4.1)	2/269 (0.7)
	PSM	16.9 ± 20.1	37/258 (14.3)	12/258 (4.7)	29/258 (11.2)	22/258 (8.5)	20/258 (7.8)	3/258 (1.2)
	*p*	0.488	0.846	0.444	0.030	0.003	0.074	0.620
BM	NSM	16.7 ± 21.9	43/335 (12.8)	9/335 (2.7)	22/335 (6.6)	13/335 (3.9)	14/335 (4.2)	3/335 (0.9)
	PSM	15.4 ± 18.0	31/192 (16.1)	12/192 (6.3)	23/192 (12.0)	16/192 (8.3)	17/192 (8.9)	2/192 (1.0)
	*p*	0.508	0.293	0.044	0.032	0.031	0.028	0.868
BL	NSM	15.1 ± 20.0	36/290 (12.4)	12/290 (4.1)	18/290 (6.2)	5/290 (1.7)	14/290 (4.8)	3/290 (1.0)
	PSM	17.6 ± 21.2	38/237 (16.0)	9/237 (3.8)	27/237 (11.4)	24/237 (10.1)	17/237 (7.2)	2/237 (0.8)
	*p*	0.184	0.234	0.842	0.034	< 0.001	0.255	0.822

*AM* apical medial, *AL* apical lateral, *MM* middle medial, *ML* middle lateral, *BM* basal medial, *BL* basal lateral, *NSM* negative surgical margin, *PSM* positive surgical margin.

On multivariate analysis, a positive core on AL biopsy (hazard ratio [HR]: 1.781, *p* = 0.008) was associated with surgical margin positivity after radical prostatectomy, in addition to BMI, biopsy Gleason grade, preoperative PSA level, and prostate volume (Table 4). After adjusting for these variables, positive AL (HR: 1.919, *p* = 0.020), BL (HR: 9.176, *p* < 0.001), BM (HR: 3.302, *p* = 0.031), and ML (HR: 2.501, *p* = 0.044) biopsies were significantly associated with positive apical, posterior, basal, and lateral surgical margins, respectively (Table 5). However, positive anterior and posterolateral margins were not associated with any specific site on prostate biopsy.

**Table 4:** Variables associated with surgical margin positivity.

	Univariate		Multivariate	
	HR (95% CI)	*p*	HR (95% CI)	*p*
Age (continuous)	1.009 (0.979–1.040)	0.574		
BMI (continuous)	1.055 (0.983–1.131)	0.135	1.078 (0.995–1.167)	0.065
Hypertension (yes vs. no)	1.229 (0.849–0.780)	0.274		
Diabetes (yes vs. no)	1.271 (0.787–2.053)	0.328		
PSA (continuous)	1.077 (1.030–1.126)	0.001	1.056 (1.004–1.111)	0.035
Prostate volume	0.987 (0.974–1.001)	0.063	0.986 (0.972–1.001)	0.072
Days from biopsy to surgery (≥ 42 days) (yes vs. no)	1.044 (0.689–1.583)	0.839		
**Biopsy Gleason grade**				
1	Reference		Reference	
2	1.344 (0.804–2.247)	0.259	1.368 (0.768–2.438)	0.288
3	1.926 (1.120–3.312)	0.018	1.780 (0.974–3.254)	0.061
4	1.967 (1.142–3.388)	0.015	1.436 (0.781–2.643)	0.244
5	5.539 (2.107–14.56)	0.001	4.565 (1.695–12.29)	0.003
Number of positive cores (continuous)	1.116 (1.038–1.200)	0.003		
Focal lesion on MR imaging (yes vs. no)	2.165 (1.483–3.162)	< 0.001		
**Location of focal lesion on MR imaging**				
Peripheral zone	Reference			
Transition zone	0.431 (0.149–1.240)	0.118		
Both	5.167 (0.565–47.23)	0.146		
Size of focal lesion on MR imaging	1.049 (0.973–1.130)	0.212		
Surgical methods (open vs. minimal invasive)	1.221 (0.840–1.775)	0.295		
**Site of biopsy positive for prostate cancer**				
AM	1.227 (0.848–1.776)	0.278		
AL	0.863 (1.280–2.711)	0.001	1.781 (1.162–2.731)	0.008
MM	1.33 (0.919–1.926)	0.130		
ML	1.586 (1.093–2.300)	0.015		
BM	1.672 (1.145–2.440)	0.008		
BL	1.551 (1.070–2.247)	0.02		

*AM* apical medial, *AL* apical lateral, *MM* middle medial, *ML* middle lateral, *BM* basal medial, *BL* basal lateral, *NSM* negative surgical margin, *PSM* positive surgical margin.

**Table 5:** The impact of site of positive biopsy for Site-specific surgical margin positivity (Adjusted by BMI, PSA, prostate volume, biopsy Gleason grade).

Site of surgical margin	Statistically significant site of biopsy (+) for prostate cancer	HR (95% CI)	*p*
Apical margin	AL	1.919 (1.108–3.34)	0.020
Posterior margin	BL	9.176 (2.706–31.12)	< 0.001
Basal margin	BM	3.302 (1.118–9.757)	0.031
Lateral margin	ML	2.501 (1.024–5.109)	0.044
Anterior margin	None		
Posterolateral margin	None		

*AM* apical medial, *AL* apical lateral, *MM* middle medial, *ML* middle lateral, *BM* basal medial, *BL* basal lateral.

## Discussion

Surgical margin status is one of the most important modifiable variables and is significantly associated with oncologic outcomes after radical prostatectomy^14^, especially in men with locally confined prostate cancer. Despite the implementation of several technical improvements, PSM after radical prostatectomy is still reported in 10–20% of patients with localized prostate cancer^15^. In this regard, novel methods for reducing PSM during radical prostatectomy are awaited. Accurately predicting surgical margin positivity after radical prostatectomy could be the first step in minimizing this outcome. In the current study, we propose an easy-to-implement and readily available novel method for predicting PSM based on positive biopsy sites. This method could help clinicians predict the surgical margin status after radical prostatectomy and minimize PSM.

In this study, PSM was observed in 31.9% of the patients. This rate seemed to be higher than that reported in previous studies. This discrepancy is believed to be due to the higher pathologic stage in patients in the current study. As mentioned previously, over 30% of patients showed locally advanced disease on pathologic examinations, which might come from the Racial differences between Asian and Western patients. On the basis of previous studies, Korean men show a higher incidence of aggressive prostate cancer than Western men^16,17^, which seems to support our findings. When considering the proportion of cases of locally advanced disease, the PSM rate of 31.9% was considered acceptable.

In the current study, only a positive AL core was significantly associated with increased PSM, regardless of the location of the surgical margin. This finding is believed to be due to the dissection technique at the AL side of the prostate. During the dissection of the AL side of the prostate, the neurovascular bundle and rhabdosphincter were pushed away from the prostate to preserve functional outcomes. In other words, apical dissection of the prostate is considered the most important procedure during radical prostatectomy because it plays a role in balancing between avoiding PSM and preventing postoperative incontinence and erectile dysfunction. Moreover, it is important to know the risk of apical PSM before surgery^18^. According to the results of the current study, the rate of a positive apical margin, which has been shown to have a higher recurrence risk than other margins^19^, was significantly increased in men with a positive AL biopsy, consistent with a previous study^20^. On the basis of these data, during nerve-sparing radical prostatectomy, apical dissection of the prostate should be carefully performed in men with a positive AL biopsy to reduce the probability of PSM. Moreover, the higher risk for PSM after nerve-sparing prostatectomy needs to be explained to patients before the surgery. These data may also be useful for MR-target biopsy, although further studies are needed for verification. In other words, positive sites on prostate biopsy could help enhance the predictive power of multiparametric MR imaging for predicting apical prostate cancer^18^.

To our knowledge, this is the first study to reveal the detailed relationship between the sites of positive prostate biopsy and the location of PSM in the final pathology. Surgeons could predict the location of PSM based on the biopsy results, and this information could be especially important for performing careful dissection during radical prostatectomy. The results of the current study suggest that apical dissection needs to be carefully performed in men with a positive AL core, as well as posterior resection in men with a positive BL core, basal resection in men with a positive BM core, lateral resection in men with a positive ML core. This seems convincing because the site of each biopsy core is located near the location of PSM. The anterior and posterolateral margins of the prostate, which are usually ligated and/or resected during prostate removal, were not associated with any site of positive prostate biopsy in the current study.

In addition to prostate biopsy-related variables, some clinically important variables, such as BMI, biopsy Gleason grade, preoperative PSA level, and prostate volume, were found to be associated with PSM after radical prostatectomy, in accordance with previous studies^4^. However, the time period between biopsy and surgery was not associated with the surgical margin status, similar to a previous report^21^. Although a period of 6 weeks between biopsy and surgery has been recommended to minimize post-biopsy adhesive changes, radical prostatectomy could be safely offered without the additional risk of PSM even if the time between biopsy and surgery was shortened to < 6 weeks; however, other perioperative outcomes need to be considered. However, interestingly, MR findings, including visible focal lesion on MR, its size and location were not significantly associated with PSM after surgery which was not in accordance with previous studies^22,23^. These findings are thought to show that detailed biopsy-related variables might be at least as important as some MR findings in predicting PSM after surgery in patients with clinically localized prostate cancer with PSA < 20 ng/mL. However, because the current study included very long period of MR-imaging, it is not easy to extract some important MR-related factors such as apparent diffusion coefficients values^23^ and the impacts of these variables cannot be evaluated in this study.

This study had several limitations, including its retrospective design. In addition, the long study period was considered to be a limitation because improvements in the surgical technique could have changed during this period. Another limitation is that the surgical margin length and Gleason score, which previously reported to increase risk of biochemical recurrence after surgery^24^, were not reported. Therefore, the severity of the surgical margin and its impacts on recurrence cannot be assessed in this study. However, to our knowledge, this study is the first to report the association between prostate biopsy results and site-specific surgical margin status on the final pathologic examination. In addition, the current study is expected to be helpful in implementing detailed surgical treatment through personalized maximal nerve preservation while reducing PSM. Although the results need to be verified in a larger study, the current study provides insights toward minimizing surgical margin positivity after radical prostatectomy in patients with locally confined prostate cancer.

## Methods

### Study population

**Figure 1:**
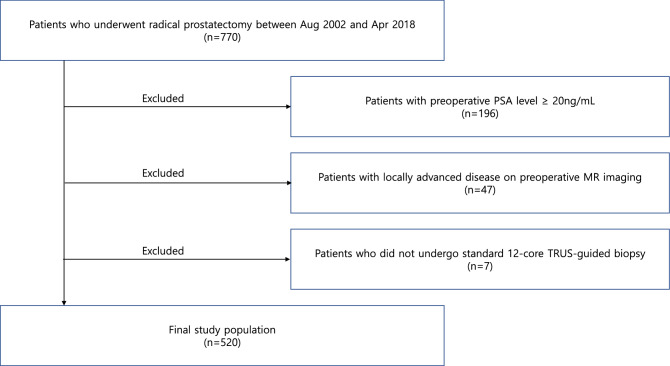
Study flow diagram.

Among 770 patients who underwent radical prostatectomy at Boramae Medical Center between August 2002 and April 2018, patients with preoperative prostate-specific antigen (PSA) level ≥ 20 ng/mL and suspected locally advanced disease, including extracapsular extension and/or seminal vesicle invasion on preoperative magnetic resonance (MR) imaging, were excluded (Fig. 1). In addition, patients who did not undergo standard 12-core transrectal ultrasound (TRUS)-guided biopsy were excluded. After the exclusion, 520 patients with preoperative PSA level < 20 ng/mL and locally confined disease on preoperative MR imaging who underwent radical prostatectomy at our institute were finally selected for the analysis. The medical records of these patients were retrospectively reviewed. The current study was approved by the institutional review board of Boramae Medical Center. We confirmed all methods were performed in accordance with the relevant guidelines and regulations.

### Patient evaluation

At our institute, standard 12-core TRUS-guided prostate biopsy is generally recommended for patients with a PSA level of ≥ 3 ng/mL. MR imaging is generally not performed without a histologic diagnosis of prostate cancer because the Korean national health insurance system does not cover the costs for MR imaging for such cases. The location of each core obtained during a 12-core standard TRUS-guided prostate biopsy was recorded. Although some proportion of patients underwent 1–3 additional core biopsy due to abnormal rectal exams and/or imaging findings, the results of these additional biopsy cores were excluded before analysis. In our institute, additional core biopsy was performed after completion of standard 12-core standard biopsy. For the analysis, the 12 sites of prostate biopsy were recategorized into 6 sites, as follows: apical medial (AM), apical lateral (AL), middle medial (MM), middle lateral (ML), basal medial (BM), and basal lateral (BL). After the histopathologic diagnosis of prostate cancer, MR imaging was routinely performed. The MR images were interpreted by radiologists specialized in urology. MR interpretation included the presence of focal lesion suspicious for prostate cancer. In addition, among 192 cases with focal lesion on MR imaging, size of focal lesion was interpreted in 86 (44.8%) cases, and location of focal lesion (peripheral zone vs. transition zone) was interpreted in 190 (99.0%) cases. Bone scans were optionally performed for intermediate- or high-risk prostate cancer patients. For these patients, radical prostatectomy was performed by 3 urologists, and the surgical method (robotic vs. open) was selected after sufficient consultation. Nerve sparing procedures was generally performed for potent and sexually active patients with low or intermediate risk prostate cancer. Specimens obtained from prostate biopsy and radical prostatectomy were examined by pathologists specialized in urology. The pathologic reports also included the location of the PSM, which was identified as follows: apex, base, anterior, posterior, lateral, or posterolateral.

### Statistical analysis

The patients were divided into 2 groups according to the surgical margin status (positive vs. negative). Baseline characteristics and pathologic characteristics are expressed as mean ± standard deviation or number with percentage. The proportion of men with PSM at specific locations (apex, base, anterior, posterior, lateral, and posterolateral) was demonstrated according to the site of positive prostate biopsy. Univariate and multivariate analyses were performed to assess the impact of the positive prostate biopsy location on the surgical margin status. In addition, multivariate analysis was performed to assess the impact of the positive prostate biopsy location on site-specific surgical margin positivity, including apical, basal, anterior, posterior, lateral, and posterolateral PSM, after adjusting for several variables. Variables with *p* values of < 0.2 in the univariate analysis were selected for the multivariate analysis, in which backward elimination methods were used. All statistical analyses were performed using IBM SPSS Statistics version 21 (IBM SPSS, Armonk, NY, USA), and *p* values of < 0.05 were considered statistically significant.

### Ethics approval

The current study was approved by the institutional review board of Boramae Medical Center.

### Consent to participate

Consent was waived by the institutional review board of Boramae Medical Center.
